# Adaptin evolution in kinetoplastids and emergence of the variant surface glycoprotein coat in African trypanosomatids

**DOI:** 10.1016/j.ympev.2013.01.002

**Published:** 2013-04

**Authors:** Paul T. Manna, Steven Kelly, Mark C. Field

**Affiliations:** a Department of Pathology, University of Cambridge, Tennis Court Road, Cambridge CB2 1QP, UK; b Oxford Centre for Integrative Systems Biology, Department of Biochemistry, University of Oxford, South Parks Road, Oxford OX1 3QU, UK

**Keywords:** Endocytosis, Variant surface glycoprotein, Adaptin, *Trypanosoma grayi*

## Abstract

The kinetoplastids are an important group of protozoa from the Excavata supergroup, and cause numerous diseases with wide environmental, economic and ecological impact. *Trypanosoma brucei*, the causative agent of human African trypanosomiasis, expresses a dense variant surface glycoprotein (VSG) coat, facilitating immune evasion *via* rapid switching and antigenic variation. Coupled to VSG switching is efficient clathrin-mediated endocytosis (CME), which removes anti-VSG antibody from the parasite surface. While the precise molecular basis for an extreme CME flux is unknown, genes encoding the AP2 complex, central to CME in most organisms, are absent from *T. brucei*, suggesting a mechanistic divergence in trypanosome CME. Here we identify the AP complex gene cohorts of all available kinetoplastid genomes and a new *Trypanosoma grayi* genome. We find multiple secondary losses of AP complexes, but that loss of AP2 is restricted to *T. brucei* and closest relatives. Further, loss of AP2 correlates precisely with the presence of VSG genes, supporting a model whereby these two adaptations may function synergistically in immune evasion.

## Graphical abstract

**Graphical abstract:**

**Figure:**
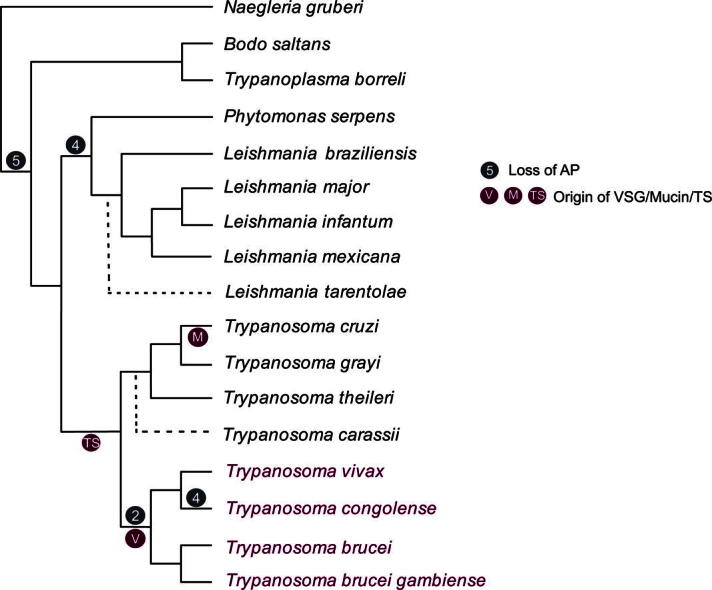

**Highlights:**

► We identify the adaptin gene cohort from currently available kinetoplastid genomes. ► Further, we present analysis of newly sequenced *Trypanosoma grayi* adaptins. ► We describe the evolutionary history of kinetoplastid adaptins. ► Kinetoplastid adaptin cohorts are compared to major surface protein families. ► Adaptin evolution and immune evasion mechanisms are correlated in salivarian trypanosomes.

## 1 Introduction

The Kinetoplastida are a protozoan class belonging to the Excavata supergroup, encompassing numerous medically and agriculturally important pathogens, as well as free-living representatives that have huge ecological impact. Multiple features indicate these organisms as highly divergent, and potentially early branching in the evolution of the eukaryotes (Cavalier-Smith, 2010). Within the kinetoplastida, the order Trypanosomatida contains many pathogens, including *Trypanosoma brucei*, *T. cruzi* and *Leishmania* spp., the causative agents of African trypanosomiasis, Chagas’ disease and leishmaniasis respectively. These parasites have evolved diverse immune evasion strategies; while *Leishmania* spp. and *T. cruzi* exploit intracellular lifestyles by invasion of host cells, *T. brucei* persists within the host bloodstream and lymphatic system, and is therefore continually exposed to both innate and adaptive immune mechanisms. The surface of mammalian infective *T. brucei* is dominated by approximately 2 × 10^7^ molecules of a single GPI-anchored variant surface glycoprotein (VSG), shielding invariant surface antigens from antibody recognition (Borst and Cross, 1982; Mehlert et al., 2002). VSG is however highly immunogenic and evasion of the host immune response is accomplished *via* antigenic variation, the periodic switching of mono-allelic expression between VSG genes (Pays, 2005). Additionally, clearance of host antibodies from the parasite surface *via* extremely rapid clathrin-dependent endocytosis of antibody bound surface proteins and antibody degradation aids immune evasion whilst host antibody titre is low and may also protect invariant epitopes from immunological recognition (O’Beirne et al., 1998; Allen et al., 2003; Engstler et al., 2007; Field and Carrington, 2009).

Intracellular vesicular transport requires both structural coat proteins and cargo adaptors functioning at discrete intracellular locations for faithful targeting (Bonifacino and Lippincott-Schwartz, 2003). Arguably the best understood cargo adaptors are the assembly polypeptide or AP complexes. Five AP complexes are known, each comprising two large subunits, one medium and one small subunit, together referred to as adaptins (Boehm and Bonifacino, 2001; Hirst et al., 2011). At least two AP complexes are intimately involved in clathrin-mediated transport pathways. The AP1 complex, comprising large β1 and γ subunits together with a medium μ1 and small σ1 subunit, sorts cargo into clathrin coated vesicles travelling between the *trans*-Golgi network and endosomes. The AP2 complex, (β2, α, μ2 and σ2 subunits) drives cargo recruitment and clathrin-mediated endocytosis (CME) at the plasma membrane (Jackson et al., 2010). The AP3 complex (β3, δ, μ3, σ3), mediates delivery of proteins including the vesicle tether VAMP7 to late endosomes/lysosomes and lysosome related organelles (Martinez-Arca et al., 2003; Dell Angelica, 2009). Whilst the AP3 β3 subunit encodes a clathrin interaction motif it appears that clathrin is largely dispensable for the known functions of AP3 (Peden et al., 2002). Comparatively little is known about the functions of either AP4 (β4, ε, μ4, σ4) or AP5 (β5, ζ, μ5, σ5), although both are likely involved in post-Golgi transport and neither appear to use clathrin (Burgos et al., 2010; Hirst et al., 2011). Uniquely, and apparently paradoxically given its huge reliance upon clathrin-mediated endocytosis, *T. brucei* has dispensed with the AP2 complex entirely, the only reported incidence of this secondary loss (Berriman et al., 2005; Field et al., 2007; Nevin and Dacks, 2009). We previously suggested that an extreme flux of CME in *T. brucei* relative to other eukaryotes, plus the high density of VSG, drove adaptation to a non-specific, rapid, AP2-independent mode of CME in African trypanosomes (Field and Carrington, 2009). Previously however, the limited number of genomes available from related taxa has made it difficult to assess whether the loss of the AP2 complex is indeed a unique feature of the African trypanosomes, and thus potentially related to their unusual surface architecture, or a more widely distributed adaptation among trypanosomatids and their closer relatives.

To explore in more detail the evolutionary relationships between extracellular parasitism, the VSG coat and AP complex evolution, we have sequenced the genome of *Trypansoma grayi* strain ANR4, a parasite with a lifestyle similar to *T. brucei*, i.e. of African location, Tsetse fly transmitted and living extracellularly in its vertebrate (crocodile) host (Minter-Goedbloed et al., 1993; Hoare, 1929). Earlier phylogenetic studies suggest that *T. grayi* is more closely related to the intracellular South American *T. cruzi* (Stevens et al., 1999) (Stevens and Gibson, 1999) (Hamilton et al., 2007) and hence *T. grayi* is a critical taxon for understanding AP complex evolution in trypanosomes. We identified the adaptin and potential major surface protein genes from *T. grayi* and all currently available trypanosomatid and closely related bodonid genomes and examined the evolutionary history of these gene families. Our analysis shows that the loss of AP2 is restricted to the African trypanosomes and correlates with emergence of the VSG coat, lending support to the concept of co-evolution of these two features.

## 2 Materials and methods

### 2.1 Genome sequence databases

Publicly available genomes included in this analysis were Trypanosoma brucei brucei 927, *Trypanosoma brucei* gambiense, *Trypanosoma congolense*, *Trypanosoma vivax*, *Trypanosoma cruzi*, *Leishmania braziliensis*, *Leishmania infantum*, *Leishmania major*, *Leishmania mexicana*, *Leishmania tarentolae*, and were all searched at TriTrypDB, (www.tritrypdb.org), while *Bodo saltans* was from geneDB (www.genedb.org) and *Naegleria gruberi* from the National Centre for Biotechnology Information (www.ncbi.nlm.nih.gov) and the Joint Genome Initiative (www.jgi.doe.gov) respectively. *Trypanosoma carassii*, *Trypanosoma theileri*, *Trypanoplasma borrelli* were part of a transcriptome project by one of us (SK) and to be published in full later, and *Phytomonas serpens* data were kindly provided by Julius Lukes (Institute for Parasitology, České Budějovice). All sequences and alignments are available from the authors on request.

### 2.2 Genome sequencing

*T. grayi* strain ANR4 genomic DNA, a kind gift of Wendy Gibson (University of Bristol), was extracted from agarose plugs using standard phenol/chloroform methods. DNA was sequenced by 91 bp paired-end Illumina sequencing at the Beijing Genomics institute (www.genomics.cn/en/). Reads were clipped based on phred score >20 using the fastx program. Read errors and ambiguous bases were then corrected using the ALLPATHS (MacCallum et al., 2009) find read errors algorithm, with two cycles of read error correction and the default settings for *k*-mer size. Duplicate reads and reads with a post-clipped length of less than 20 nucleotides were discarded using custom Perl scripts. The clipped, corrected and filtered reads were then assembled using Velvet (Zerbino and Birney, 2008) and multiple *k*mer sizes (*k*mer = 31, 41, 51 and 61). The resulting contigs from all assemblies were then post-assembled using CAP3 (Huang and Madan, 1999) to yield a final genome assembly. *k*mer frequency analysis of filtered, clipped and corrected reads yielded an estimated genome size of 29,355,514 bases, at approximately 48*x* coverage, highly consistent with the haploid genome size estimates for other African trypanosomes.

### 2.3 Homology searches

Adaptin searches were performed with BLAST using *Homo sapiens* sequences as queries against genome sequence data from all available trypanosomatids and bodonids as well as the free living heterolobosid excavate *Naegleria gruberi* (Table S1). In cases where orthologs were not found, further searches were carried out using sequences from phylogenetically close organisms. All retrieved sequences were then verified by reciprocal BLAST analysis against the *H. sapiens* genome database. Major surface protein family searches were carried out with BLAST using multiple representatives of each protein family (Table S1) and genes were assigned as present based upon BLAST score (*e* < 1 × 10^−5^) and reciprocal BLAST retrieving members of the target gene family.

### 2.4 Phylogenetic reconstruction

Sequences were aligned using MUSCLE (Edgar, 2004) and edited manually to remove poorly conserved regions. For each protein family the optimal substitution model was assessed (ProtTest3 (Abascal et al., 2005)) and phylogenetic trees were generated using Bayesian (MrBayes, (Ronquist and Huelsenbeck, 2003)) and maximum likelihood (RaxML, (Stamatakis, 2006), and PhyML, (Guindon and Gascuel, 2003)) approaches. For maximum likelihood calculations, the best fitting amino acid substitution models and parameters according to ProtTest3 were; for the γ, α, δ, ε adaptin family Le and Gascuel (LG) substitution model (Le and Gascuel, 2008) with gamma (G) correction of 2.08, for β adaptins LG modified with the observed amino acid frequencies (+F)+G (1.35), for μ adaptins LG +F,+I (0.03)+G (2.54), for the σ adaptins Jones, Taylor and Thornton model (JTT) (Jones et al., 1992) +F +I (0.11)+G (1.65) and for the HSP90 gene family LG + G (0.47). PhyML was run *via* the South of France Bioinformatics Platform web server (www.atgc-montpellier.fr/phyml/). RaxML was run via the Cyberinfrastructure for Phylogenetic Research (CIPRES) Science Gateway web server (www.phylo.org). Bayesian tree topologies and posterior probabilities were calculated using MrBayes version 3.1.2, analyses were run for 1 × 10^6^ generations, removing all trees before a plateau, established by graphical estimation. MrBayes was run on CamGRID. All adaptin trees were rooted at AP3 (Hirst et al., 2011). New sequence data have been submitted to GenBank, and accession numbers for all sequences included in the analysis are given in Table S1.

## 3 Results

### 3.1 Representation of AP complexes in kinetoplastids

**Supplementary Fig. 1:**
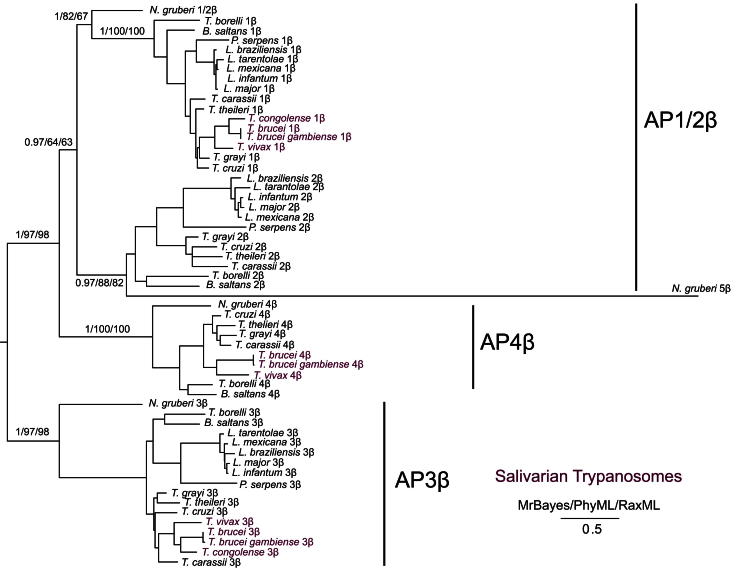
Phylogenetic reconstruction of Kinetoplastida β-adaptins. The tree shown is the best bayesian topology. Branch support for important clades is given according to the key. Note the absence of salivarian sequences in the AP2β clade and the restriction of AP5β to *N. gruberi*.

**Supplementary Fig. 2:**
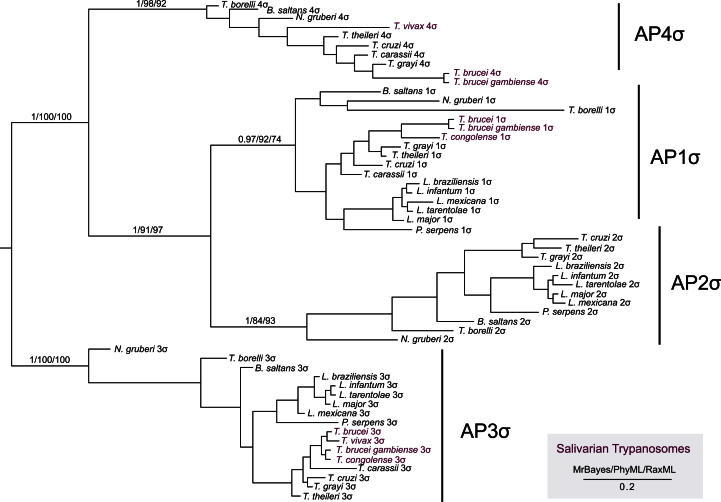
Phylogenetic reconstruction of Kinetoplastida σ-adaptins. The tree shown is the best bayesian topology with branch support for important clades given according to the key. Again no salivarian sequences corresponding to AP2σ were identified. Also note a duplication of AP4σ in *N. gruberi*.

**Supplementary Fig. 3:**
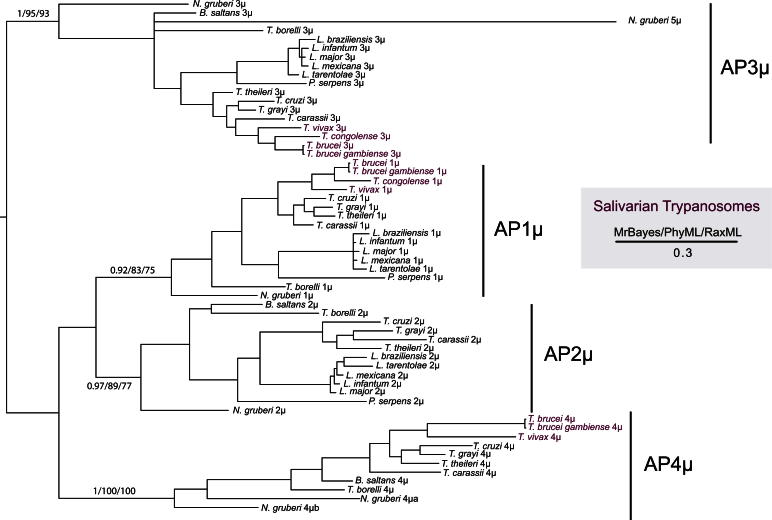
Phylogenetic reconstruction of Kinetoplastida μ-adaptins. The tree shown is the best bayesian topology with branch support for important clades given according to the key. No AP2μ sequences were identified in the salivaria. AP5μ is restricted to *N. gruberi*, which also shows a further duplication of the AP4 specific μ subunit.

**Fig. 1:**
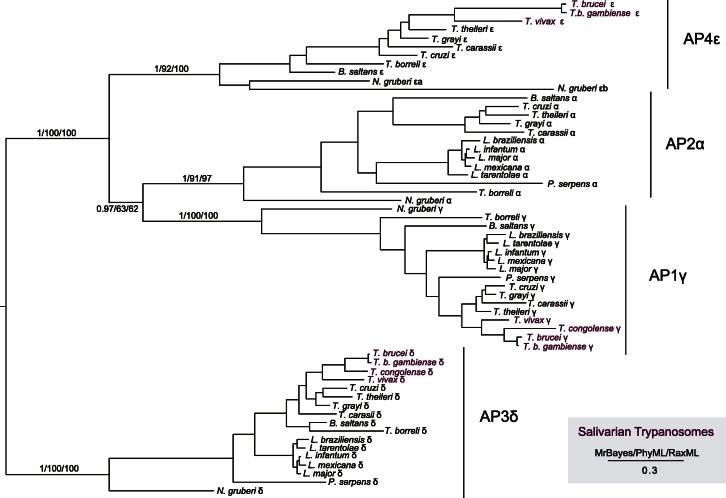
Phylogenetic reconstruction of the unique adaptin family γ, α, δ and ε large subunit. The tree shown is the best Bayesian topology with branch support for important nodes indicated. Identified adaptins group reliably by predicted AP complex (see methods) with clades indicated by vertical bars. Note the absence of the AP2 complex α-subunit from all salivarian trypanosomes.

Genome searches yielded well conserved adaptin subunit homologues from all of the organisms examined. Phylogenetic analysis of the adaptin subunits produced well supported clades, which in all cases corresponded to AP complexes 1 through 4, with AP5 subunits restricted to *N. gruberi* as previously suggested, but extending this observation across the whole of the trypanosomatids (Figs. 1, S1–S3) (Hirst et al., 2011). In line with studies of other eukaryotic lineages, principally the chromalveolates (Field et al., 2007; Nevin and Dacks, 2009), a repeated loss of adaptins and entire AP complexes was seen across the trypanosomatids, with loss of AP4 from the *Phytomonas* and *Leishmania* clade as well as the salivarian trypanosome *T. congolense*. This is likely a result of at least two independent events, one at the base of the *Phytomonas*/*Leishmania* clade and a second more recent event following speciation of *T. congolense*. We were unable to identify a sequence corresponding to μ1 adaptin from *B. saltans*; however, we suggest this may be a result of incomplete data as the remaining AP1 subunits were confidently identified.

The AP2 complex is absent entirely from all salivarian (*T. brucei* clade) trypanosomes, confirming our earlier observations (Morgan et al., 2002). Significantly, AP2 was found to be present in all other organisms considered here, including *T. grayi*, *T. carassii* and *T. theileri*, arguing against loss of AP2 and adaptation within the early endocytic pathway as a prerequisite to an extracellular parasitic lifestyle.

### 3.2 Major surface antigen families

To assess the relationship between AP complement and the surface coat, and to perhaps reveal some details about the immune evasion strategy of *T. grayi* we also searched each genome for the presence of the major trypanosomatid surface protein families, VSG, amastins, *trans*-sialidase, mucins and gp63. *Trans*-sialidase is a unique cell surface enzyme responsible for the modification of surface proteins in trypanosomes, and implicated in virulence and invasion pathways (Schenkman et al., 1991). Amastins are transmembrane cell surface proteins conserved across the trypanosomatids, *albeit* at greatly differing copy numbers, with currently unknown function (Teixeira et al., 1994; Jackson, 2010). The latter two protein families are the predominant surface glycoproteins from *T. cruzi* and *Leishmania* respectively, and while they are large paralogous families with complex expression patterns, they are not associated with classical antigenic variation (Acosta-Serrano et al., 2001) (Yao, 2010). Furthermore, gp63 is widely distributed, and this was confirmed by our searches which detected sequences encoding gp63 in all of the taxa analysed. Significantly this extends to *N. gruberi*, indicating that gp63 predates the kinetoplastida, and gp63 is in fact likely pan-eukaryotic (data not shown). Another large family of cell surface proteins identified in *T. cruzi* are the mucin associated surface proteins (MASPs), which appear to be GPI-anchored cell surface proteins (Bartholomeu et al., 2009). Unfortunately, attempts to assign sequences corresponding to MASPs were hampered by low complexity and high sequence divergence within this gene family; as a result we were unable to assign MASP sequences with confidence and have therefore been unable to include this gene family in the present analysis. *Trans*-sialidase sequences were found in all trypanosomes, but not in the bodonid *B. saltans* or *T. borreli*, or in the non-trypanosome trypanosomatids (*Leishmania* spp., *P. serpens*) suggesting an origin at the root of the trypanosomes. Significantly, the *T. cruzi* mucin family were found only in that taxon. Whilst the surface coat of *T. carassii* is biochemically similar to the mucin coat of *T. cruzi* (Lischke et al., 2000), homology searches with either type 1 or type 2 mucins from *T. cruzi* did not retrieve high scoring sequences from any other stercorarian trypanosome, suggesting sequence, if not functional divergence in the cell surface coats of these parasites. Finally, VSG was found only in the salivarian trypanosomes.

### 3.3 HSP90 phylogeny of kinetoplastids and coevolution of surface and AP complexes

**Fig. 2:**
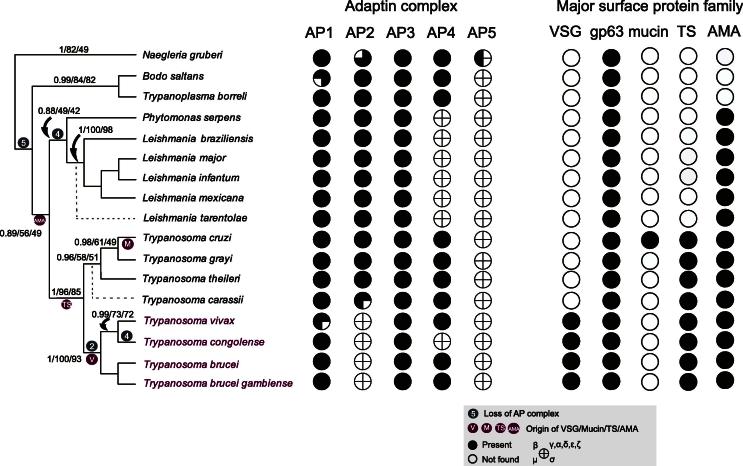
Reconstruction of AP complex evolutionary history in kinetoplastids. Left side: points of presumed AP complex loss are shown superimposed upon a cladogram based upon the cytosolic HSP90 protein family. Note multiple losses of the AP4 complex and the single loss of AP2 at the base of the extant salivarian trypanosomes, coincident with the emergence of the VSG coat (purple). Dotted lines represent suggested placement of *T. carassii* and *L. tarentolae* based on previously reported 18S rRNA gene sequences (Stevens et al., 1999; Votypka et al., 2010) due to the inability to detect cytosolic HSP90 genes. Right side: Coulson plot of distribution of adaptin and surface antigen (TS: trans-sialidase, AMA: amastin) gene families; genes are represented as columns and taxa as rows. Adaptin subunits are assigned based upon phylogenetic positioning and reciprocal blast analysis. Surface protein families were identified by reciprocal blast (see methods).

To anchor the evolutionary history of the kinetoplastids, a phylogenetic tree was constructed based upon highly conserved cytosolic HSP90 protein sequences (Simpson et al., 2002; Stechmann and Cavalier-Smith, 2003) (Fig. 2). No sequences corresponding to a cytosolic HSP90 gene were identified from either *L. tarentole* or *T. carassii*, therefore these were positioned according to previously reported 18S rRNA gene sequence phylogenies (Stevens et al., 1999; Votypka et al., 2010) (Fig. 2). Against this phylogeny we mapped adaptin complex and surface antigen distributions in order to ascertain points of invention and secondary loss (Fig. 2). AP2 appears to have been lost just once during trypanosomatid evolution, in the common ancestor of extant salivarian trypanosomes, and significantly, at present resolution, this is coincident in the phylogeny as the origin of VSG. The current data set cannot resolve if VSG appearance predated AP2 loss or *vice versa*, but it is clear that both events occurred within a relatively short evolutionary period. Secondary loss of AP4 did not correlate with a change to the surface coat, nor was there an accompanying change to the AP complement with the origin in *T. cruzi* mucins, Trypanosomatid specific *trans*-sialidases or *pan* trypanosomatid amastins.

## 4 Discussion

One of the major motivations for genome sequencing of pathogenic organisms is to gain insights into the virulence mechanisms that these organisms employ. The trypanosomatids exhibit a huge range of life styles, and include species that exploit intracellular mechanisms for immune evasion, as well as extracellular life styles. These latter strategies, apparently restricted to African trypanosomes, require specific adaptations to avoid killing by the host innate and acquired immune responses. In the case of *T. brucei* it is well established that the VSG coat is the primary means of defence, but that this is augmented by an efficient antibody cleaning mechanism (O’Beirne et al., 1998; Allen et al., 2003; Engstler et al., 2007; Field and Carrington, 2009). It is also possible, though not proven, that this cleaning mechanism is equally important for protection against responses towards invariant epitopes, for example the invariant surface glycoproteins (Ziegelbauer and Overath, 1993; Chung et al., 2004). Significantly, a divergent African trypanosome, *T. grayi*, is also reported to dwell within the host bloodstream, but few molecular details are available for this organism (Hoare, 1929). Here we set out to address two questions; does modification to the early endocytic pathway correlate with changes to immune evasion mechanisms, and what evasion strategy does *T. grayi* employ? We approached the former by interrogation of sequence databases for 15 kinetoplastid species and the latter by shotgun sequencing of the *T. grayi* genome.

We found multiple examples of losses of the adaptin complexes. Specifically we found that all kinetoplastids lack AP5, a recently discovered complex involved in endosomal sorting (Hirst et al., 2011). As AP5 appears to mediate trafficking of the cation independent mannose-6-phosphate receptor, a specialised lysosomal pathway that is absent from the trypanosomes, there is good rationale for this loss. However, the alternate interpretation, that the eukaryotic root sits between kinetoplastids and the heterolobosids cannot be rigorously excluded (Cavalier-Smith, 2010), and would imply that AP5 arose after separation of kinetoplastids from the main eukaryotic lineage. Further, *Leishmania* and *Phytomonas*, which form a monophyletic superclade, all lack AP4. This complex localises to the *trans*-Golgi network, but is expressed at low levels and hence may not have a major role in post-Golgi transport. The only known cargo is the amyloid precursor protein, where AP4 appears to mediate selective transport to the endosome rather than exocytosis (Burgos et al., 2010). Similarly absence of AP4 from *T. congolense* suggests this pathway has been lost from this taxon more recently. We presume this reflects a simplification in post-Golgi transport events, but this is clearly not associated with a specific change to the immune evasion mechanism or the surface antigen coat. Indeed, no correlation was found between the emergence of any surface protein families excepting VSG and AP complex loss.

Multiple explanations for a relationship between loss of AP2 and emergence of VSG are possible. Firstly, loss of AP2 mediated sorting of cargoes into endocytic pits can be viewed as a response to the homogeneity of the VSG coat, the absence of a cytoplasmic domain from VSG precluding AP2-mediated concentration into endocytic pits. Additionally, as AP2 removal from CCVs requires a complex phosphorylation/dephosphorylation cycle (Semerdjieva et al., 2008), the presence of AP2 may decrease the maximal possible endocytic flux; loss of AP2 after VSG emerged may represent an advantageous adaptation. An alternative hypothesis would see the emergence of fast and non-selective endocytosis as a primary immune evasion strategy, aiding antibody clearance by rapid removal of surface immune complexes. Indeed, increased hydrodynamic forces acting upon VSG-antibody complexes compared to VSG alone leads to concentration towards the cell posterior and selective endocytosis (Engstler et al., 2007). The presence of this system may have been facilitatory for the emergence of a sophisticated VSG-based antigenic variation system. A recent analysis of VSGs across salivarian trypanosomes suggests that antigenic variation, although common between these organisms, differs in its underlying mechanism between *T. brucei* and *T. congolense* and the earlier diverging *T. vivax*, suggesting that their common ancestor possessed a smaller VSG cohort (Jackson et al., 2012). Although it is unclear if the ancestor already had antigenic variation, it is likely that it lacked AP2 and presumably shared the endocytic adaptations seen in extant salivarian trypanosomes. Whilst the current study is restricted to a small number of genes of known function in membrane trafficking it is important to note that there are likely other adaptations present within the bloodstream forms of the African trypanosomes that contribute to rapid clearance of cell surface VSG. One possible example is the unusual form of the GPI anchor in this life cycle stage; for example the fatty acyl chains are entirely myristic acid (Ferguson et al., 1985, 1988), an adaptation believed to be restricted to the trypanosomes expressing VSG, and with no known function.

The limited analysis of *T. grayi* sequences contained herein supports its position as a sister taxon to *T. cruzi* (Stevens et al., 1999; Stevens and Gibson, 1999). However a full assessment of the phylogenetic positioning of this organism, for example utilizing large concatenated gene datasets is beyond the scope of the current work. It is hoped however, that the genome sequence data generated will be of use in future studies seeking to address this issue, as well as the biology of *T. grayi* in general. The absence of detectable mucin genes however suggests that the parasite is unlikely to exploit similar niches as the South American trypanosome, while the ease of isolation from host blood indicates a significant presence in the bloodstream. Again, the absence of VSG suggests a distinct mechanism for immune evasion and more detailed analysis of the predicted surface proteome of *T. grayi* is in progress.

## Appendix A Supplementary material

Supplementary Table 1 Accession numbers of all query sequences and blast hits used in this analysis from publicly available genome databases, and the newly reported sequences from *T. grayi*.
